# Complete mitochondrial genome of mouthbrooding fighting fish (*Betta pi*) compared with bubble nesting fighting fish (*B. splendens*)

**DOI:** 10.1080/23802359.2017.1413294

**Published:** 2017-12-12

**Authors:** Ornjira Prakhongcheep, Narongrit Muangmai, Surin Peyachoknagul, Kornsorn Srikulnath

**Affiliations:** a Laboratory of Animal Cytogenetics and Comparative Genomics (ACCG), Department of Genetics Faculty of Science, Kasetsart University, Bangkok, Thailand;; b Animal Breeding and Genetics Consortium of Kasetsart University (ABG-KU), Bangkok, Thailand;; c Center for Advanced Studies in Tropical Natural Resources, National Research University-Kasetsart University (CASTNAR, NRU-KU) Kasetsart University, Bangkok, Thailand;; d Department of Fishery Biology Faculty of Fisheries, Kasetsart University, Bangkok, Thailand;; e Department of Biology Faculty of Science, Naresuan University, Phitsanulok, Thailand;; f Center of Excellence on Agricultural Biotechnology: (AG-BIO/PERDO-CHE), Bangkok, Thailand

**Keywords:** Mitochondrial genome, fighting fish, *Betta pi*, *Betta splendens*

## Abstract

*Betta pi* is the largest species of mouthbrooding fighting fish, while *B. splendens* is a globally ornamental bubble nesting fish. Complete mitochondrial genomes (mitogenomes) of wild individuals of *B. pi* and *B. splendens* were determined. The mitogenome sequences were 16,521 and 16,980 base pair in length, containing 37 genes with gene order identical to most teleost mitogenomes. Overall A + T content was 57.72% for *B. pi* and 61.92% for *B. splendens*. Phylogenetic analysis showed that *B. pi* and *B. splendens* were highly supported monophyletic clades. Our results will facilitate further genetic studies, including mitochondrial variations and population structure of fighting fishes.

Fighting fish in the genus *Betta* comprise the largest group in Anabantoidei. They are separated into two groups as mouthbrooders and bubble nesting (Ruber et al. 2004; Chailertrit et al. 2014). *Betta pi* is the largest mouthbrooding fighting fish species, while *B. splendens* is a globally popular ornamental bubble nesting fish with brilliant and striking colour variations. There are many fighting fish in local aquariums and pet shops but through loss of their natural habitat wild populations are in decline. The study of genetic diversity in fighting fishes thus provides important information for prospective breeding and conservation management. Here, we determined complete mitochondrial genomes (mitogenomes) of *B. pi* and *B. splendens* collected from Narathiwat (6.4255°N, 101.8253°E) (No. KUMF6447) and Pathum Thani Provinces (14.0208°N, 100.5250°E) (No. KUMF6446), respectively, and stored in Kasetsart University Museum of Fisheries (Natural History). Whole genomic DNA was extracted in accordance with the standard salting-out protocol (Supikamolseni et al. 2015). Polymerase chain reaction (PCR) amplifications were performed using universal mitogenome primers (Mauro et al. 2004), and specific primers for both *Betta* species were developed to amplify the remaining parts of the genome. All PCR products were sequenced by the DNA sequencing service of First Base Laboratories Sdn Bhd (Selangor, Malaysia), and annotation was then performed following Srikulnath et al. (2012).

The complete mitogenome sequences consisted of 16,521 bp for *B. pi* (GenBank accession no. AB920288) and 16,980 bp for *B. splendens* (AB571120). Both mitogenomes contained 37 genes, and a control region (Table 1). Gene arrangement patterns were identical to those of teleosts (Miya et al. 2013). Four conserved sequence blocks: CSB-D, CSB-1, CSB-2 and CSB-3 found in the control region of teleost mitogenomes were also found in *B. pi* and *B. splendens* (Lee and Kocher 1995). However, 19 bp insertion was observed in CSB-1 of *B. pi*. A tandem repeat was identified at positions 15,681–15,724 bp in *B. pi*. Moreover, in *B. splendens*, two tandem repeats were identified at positions 15,793–16,150 bp and 16,906–16,971 bp. This differed from the control region structure of the *B. splendens* mitogenome (KR527219) which had three tandem repeats (Song et al. 2016), suggesting that the control region in *B. splendens* varies between individuals and might be applicable for population genetic study. Comparison of overall nucleotide diversity among *B. pi*, *B. splendens* (AB571120) and *B. splendens* (KR527219) mitogenomes was determined at 13.92%. Both *B. splendens* individuals showed 0.64% nucleotide divergence, suggesting intra-specific sequence diversity. The phylogenetic tree was constructed based on concatenated twelve protein-coding genes without *ND6* of 16 teleosts with fighting fishes, using Bayesian inference with MrBayes version 3.2.6 (Huelsenbeck and Ronquist 2001). *B. splendens* (AB571120) and *B. splendens* (KR527219) as a sister clade formed a monophyletic group with *B. pi*. The fighting fish clade was close to the *Macropodus* clade, confirming results from previous studies (Ruber et al. 2004) (Figure 1). These complete mitogenomes will increase understanding of the evolutionary processes and diversification of the fighting fish group and facilitate more thorough analyses.

**Figure 1.**
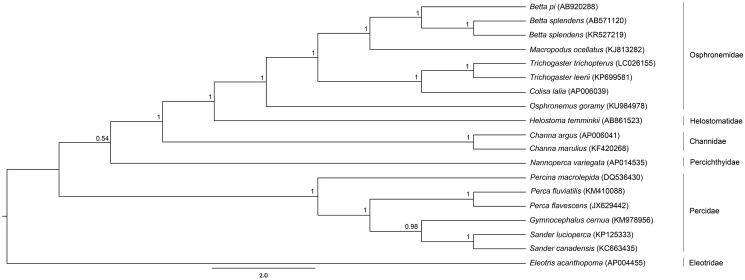
Phylogenetic relationships among concatenated mitochondrial twelve protein-coding genes without *ND6* sequences of 19 mitochondrial genomes including *Eleotris acanthopoma* as the outgroup inferred using Bayesian inference analysis. The complete mitochondrial genome sequence was downloaded from GenBank. The accession number is indicated in parentheses after the scientific name of each species. Support values at each node are Bayesian posterior probabilities. The branch-lengths represent number of nucleotide substitutions per site.

**Table 1.** Comparison of nucleotide length, AT content, start codon, stop codon and nucleotide sequence diversity between the three complete mitochondrial genomes of *Betta pi (AB920288)*, *B. splendens* (AB571120) and *B. splendens* (KR527219).

	Sequence length (bp)			AT content (%)			Start codon			Stop codon			Nucleotide sequence diversity (%)^b^		
Gene	BPI	BSP1	BSP2	BPI	BSP1	BSP2	BPI	BSP1	BSP2	BPI	BSP1	BSP2	BPI/BSP1 (%)	BPI/BSP2 (%)	BSP1/BSP2 (%)
12S rRNA	955	956	960	56.75	56.59	58.84	–	–	–	–	–	–	13.24	13.45	2.01
16S rRNA	1676	1701	1690	57.58	59.53	59.47	–	–	–	–	–	–	13.50	13.70	0.30
*ND1*	975	975	975	58.26	61.64	61.95	ATG	ATT	ATT	TAA	TAA	TAA	23.79	21.23	3.18
*ND2*	1046	1046	1046	57.84	61.00	61.00	ATG	ATG	ATG	TA+^a^	TA+^a^	TA+^a^	23.33	23.14	0.19
*COI*	1560	1560	1560	54.81	59.94	59.94	GTG	GTG	GTG	AGG	AGG	AGG	19.94	19.74	0.32
*COII*	691	691	691	58.76	61.22	61.08	ATG	ATG	ATG	T++^a^	T++^a^	T++^a^	22.43	22.43	0.14
*ATP8*	168	168	168	60.12	69.64	69.65	ATG	GTG	GTG	TAA	TAA	TAA	30.95	30.95	0.00
*ATP6*	683	684	702	57.25	65.45	64.96	ATG	ATG	ATG	TA+^a^	TA+^a^	TAA	22.25	22.40	0.59
*COIII*	786	798	786	54.20	59.65	59.41	ATG	ATG	ATG	TAA	TAA	TAA	18.58	18.70	0.13
*ND3*	349	349	349	56.73	59.03	59.03	ATG	ATG	ATG	T++^a^	T++^a^	T++^a^	21.84	20.98	0.86
*ND4L*	297	297	297	55.22	57.58	57.58	ATG	ATG	ATG	TAA	TAA	TAA	20.88	20.88	0.00
*ND4*	1381	1381	1381	57.86	62.42	62.64	ATG	ATG	ATG	T++^a^	T++^a^	T++^a^	22.30	22.23	0.29
*ND5*	1846	1851	1863	59.15	63.10	63.12	ATG	ATA	GTG	T++^a^	TAA	TAA	29.27	29.38	0.11
*ND6*	522	522	522	56.90	63.10	63.03	ATG	ATG	ATG	TAA	TAA	T++^a^	24.76	24.76	0.00
*CytB*	1156	1147	1147	56.84	62.12	62.34	ATG	ATG	ATG	T++^a^	T++^a^	T++^a^	22.67	22.84	0.35
Control region	847	1199	1327	64.58	74.71	77.62	–	–	–	–	–	–	22.54	22.54	0.24
Entire genome	16521	16980	17099	57.72	61.91	62.13							20.61	20.49	0.64

^a^ TAA stop codon is completed by the addition of 3’A residues to mRNA.

^b^ *Betta pi*: BPI; *B. splendens* (AB571120): BSP1; and *B. splendens* (KR527219): BSP2.
